# Practical Notes on Diagnosis and Treatment

**Published:** 1910-02-05

**Authors:** 


### Practical Notes on Diagnosis and Treatment.

**Anæsthetic Difficulties.**

In children especially, a powerful stimulus can be applied to the respiratory centre by the simple device of stretching the sphincter ani.

**Injuries of the Eyelids.**

First ascertain if there be any abrasion of the cornea, for, if so, on no account must lead lotion be applied, lest a dense white deposit of lead carbonate be left which seriously impairs the vision.

**Prophylaxis against Tetanus.**

Wounds contaminated with earth or otherwise thought liable to be infected with tetanus, should be thoroughly washed with peroxide of hydrogen. If possible the wound should be laid open by incision, so as to expose its depths to the air. There is but little chance of the tetanus bacillus growing on mere superficial abrasions.

**Burns.**

Before attempting to deal with any considerable burn, especially in a child, it is wise first to administer chloroform and stimulants. Then the clothes and dirt can be properly removed, the surface cleansed, and a picric acid or other dressing applied. Speed is a very desirable factor, so that the child may be put to bed as quickly as possible, and the shock combatted by warmth. The first dressing should not be disturbed for two or three days.

**Acute Bronchitis in Children.**

The steam-kettle has deservedly fallen into disuse as a routine proceeding, but it has its uses where there is a dry cough and the stethoscope discloses many dry sounds. Under no circumstances should the steaming be continuous or the bed enveloped in a tent. The full benefit will be obtained by allowing the steamer to play over the bed from the far end for fifteen minutes at a time. Fomentations are much to be preferred to poultices, as, being more easily prepared, they are more likely to be applied hot. Fresh air is more important than medicine, and the room must not be filled with anxious friends, who simply use up the atmosphere.—*Dr. G. Sutherland.*

**Neuralgia.**

Apart from endeavouring to treat the cause good results may be expected from a combination of quinine and hydrobromic acid.

**Anodyne Liniment.**

Neuralgic pains may often be relieved by the application of chloroform liniment to which has been added menthol gr. 60 to the ounce.

**Leucorrhœa.**

A useful douche in cases of leucorrhœa is one consisting of one grain of hydrochlorate of quinine dissolved in warm boric acid solution. The drug may also be employed in the form of pessaries two or three grains in each, with a glycogelatine basis; in this hazeline may be used instead of water.

**Opium Poisoning.**

The value of permanganate of potassium as an antidote to morphia has been amply demonstrated. It is recommended to give about 6 grains of antidote in 4 ozs. of water for each ounce of laudanum taken and to wash out the stomach two or three times with a weaker solution at intervals of half an hour.—*Dr. Luff.*

**Continued Pyrexia.**

In diagnosing a case of continued fever, not presenting any typical signs or symptoms, one should pass in review the following conditions: Tuberculosis, enteric, broncho-pneumonia, infective endocarditis, suppurative pylephlebitis, influenza, septicæmia, appendicitis, pelvic peritonitis, cirrhosis of the liver, Hodgkin's disease, and syphilitic fever.

**Rheumatoid Arthritis.**

Pain in the knee-joint yields more effectually to the heat from an improvised sand bath than from the intermittent applications of hot flannels. Sand, well sifted, should be baked in a biscuit-tin, and then, having arranged a blanket and rubber sheet under the knee, the sand should be poured out over the knee and packed underneath as well. The whole is covered with another folded blanket.

